# (Bio)Nanotechnology in Food Science—Food Packaging

**DOI:** 10.3390/nano11020292

**Published:** 2021-01-22

**Authors:** Mateja Primožič, Željko Knez, Maja Leitgeb

**Affiliations:** 1 Faculty of Chemistry and Chemical Engineering, University of Maribor, Smetanova 17, 2000 Maribor, Slovenia; mateja.primozic@um.si (M.P.); zeljko.knez@um.si (Ž.K.); 2 Faculty of Medicine, University of Maribor, Taborska ulica 8, 2000 Maribor, Slovenia

**Keywords:** nanotechnology, (bio)nanotechnology, food, food packaging, improved packaging, active packaging, smart packaging

## Abstract

**Background:** Bionanotechnology, as a tool for incorporation of biological molecules into nanoartifacts, is gaining more and more importance in the field of food packaging. It offers an advanced expectation of food packaging that can ensure longer shelf life of products and safer packaging with improved food quality and traceability. **Scope and approach:** This review recent focuses on advances in food nanopackaging, including bio-based, improved, active, and smart packaging. Special emphasis is placed on bio-based packaging, including biodegradable packaging and biocompatible packaging, which presents an alternative to most commonly used non-degradable polymer materials. Safety and environmental concerns of (bio)nanotechnology implementation in food packaging were also discussed including new EU directives. **Conclusions:** The use of nanoparticles and nanocomposites in food packaging increases the mechanical strength and properties of the water and oxygen barrier of packaging and may provide other benefits such as antimicrobial activity and light-blocking properties. Concerns about the migration of nanoparticles from packaging to food have been expressed, but migration tests and risk assessment are unclear. Presumed toxicity, lack of additional data from clinical trials and risk assessment studies limit the use of nanomaterials in the food packaging sector. Therefore, an assessment of benefits and risks must be defined.

## 1. Introduction

In recent decades, (bio)nanotechnology has become increasingly important as an appealing technology for the food industry. Nanotechnology is a discipline composed of several fields of technology, and serves as a tool for creating, studying phenomena, or manipulating matter in nanoscale dimensions [1]. Development of novel nanomaterials makes possible to improve food quality and safety, crop growth, and monitoring environmental conditions [2]. The obtained materials have unique properties such as high surface-to-volume ratio and their other physiochemical properties such as color, solubility, strength, diffusivity, toxicity, magnetic, optical, thermodynamic properties, etc. are improved [3,4]. Therefore, nanotechnology has brought a new industrial revolution as it offers a wide range of possibilities for the development and use of structures, materials, or systems with new or improved properties in various fields such as agriculture, food and medicine, etc. Besides, it is also one of the fastest growing fields in agriculture and food sector. Bionanotechnology is a tool for integrating biological molecules into nanostructures [5]. The growing desire of consumers for quality food and awareness of a healthy lifestyle is encouraging researchers to find a way to improve food quality while least compromising nutrition product value. Therefore, new studies are focused on developing novel methods, techniques, and procedures for the purpose of processing, packaging, functionalization, and quality control implementation of food, and also the for nutraceutical products delivery system [6]. Recently, the demand for nanoparticle-based materials for the different applications e.g., food industry has increased, especially in the EU. The European nanomaterials market generated more than $2.5 million in 2015 and is expected to reach around $9 million in revenue by 2022. Many of these materials contain essential elements, some of them non-toxic [7] that can be stable at high pressures and temperatures [8,9]. The improved properties of nanomaterials have enabled and prompted the development of technologies that extend the shelf life and freshness of products, the design of methods and tools for rapid in situ analysis and the production of environmentally safe and degradable food packaging. With the use of nanotechnology, remarkable lifestyle improvements can be achieved. Nanomaterials can contribute to the development of improved products, wealth, health, and quality. Besides, this could also reduce the impact on the environment due to their nano size [10]. Nevertheless, the usage of nanotechnology in food packaging is still in the infancy stage. Nevertheless, the use of different functional nanomaterials can improve materials for packaging, the development and application of these nanomaterials is increasing presently also in the packaging industry for food increasing the shelf life and safety of packaged food [11].

Using nanotechnology, the nanoparticles can be incorporated in film to form nanofilm that could increase certain gases permeability with the aim to reduce the concentration of harmful gases, such as carbon dioxide (CO_2_) or oxygen (O_2_), which negative affect the foods shelf life and can be also used as barrier materials to prevent spoilage by microbes [12,13,14]. Since, the most widely used materials in food packaging industries are plastic polymers which are non-biodegradable and represent a serious threat to human and the environment [15], scientific research is also focused on synthesis of edible coatings and films from bionanocomposites or employed as matrixes for incorporating antimicrobial nanoparticles to prolong shelf life and enhance storage quality of fruit and vegetables [16]. With the use of biomaterials, packaging materials may be reduced and at the same time the tremendous problem of waste may be solved. Additionally, biomaterials can contribute to prolongation of the shelf life, as well as safety and quality of food during the distribution, storage and consumption period may be extended [17,18]. However, the use of biomaterials in food packaging is still very limited.

**Figure 1:**
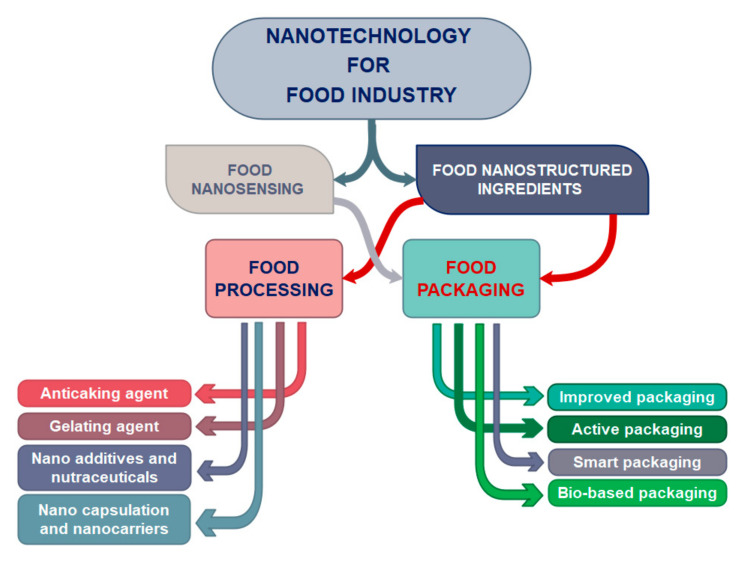
Application of (bio)nanotechnology in different fields of the food industry.

The application of nanotechnology in food industry can be divided into two major groups: food nanosensing and food nanostructured ingredients [3]. Nanostructured food ingredients cover a wide range including food processing and food packaging whereas the field of food nanosensing provides improved food quality and safety (Figure 1).

In the field of food processing, nanostructures and nanostructured materials can be used as: (a) food additives and carriers for smart delivery of nutrients; to improve nutritional value of food, (b) anticaking agents; to improve the consistency of food and to prevent the lump formation, (c) gelating agents; to improve the food texture and (d) nanocapsules and nanocarriers; to protect aroma, flavor and other ingredients in food. While, in the field of food nano-packaging, improved packaging, active packaging, smart packaging and bio-based packaging are considered.

In this review, the role of (bio)nanotechnology in food science with emphasis on application of (bio)nanomaterials in the field of packaging is summarized and also some negative acts associated with application of nanotechnology in the field of food application are discussed. Future perspective and aspects of nanotechnology in food industry are included.

## 2. Food Packaging

Food packaging is one of the most critical steps in terms of food safety. The purpose of food packaging is primarily to prevent spoilage and contamination, increase sensitivity by enabling enzyme activity, and reduce weight loss [2]. In functional foods bioactive component often gets degraded and inactivated due to the hostile environment and consequently the shelf life of the product is shortened. The use of nanostructured or nanomodified materials is a promising way to prolong and ensure the shelf life of a food product [19].

**Figure 2:**
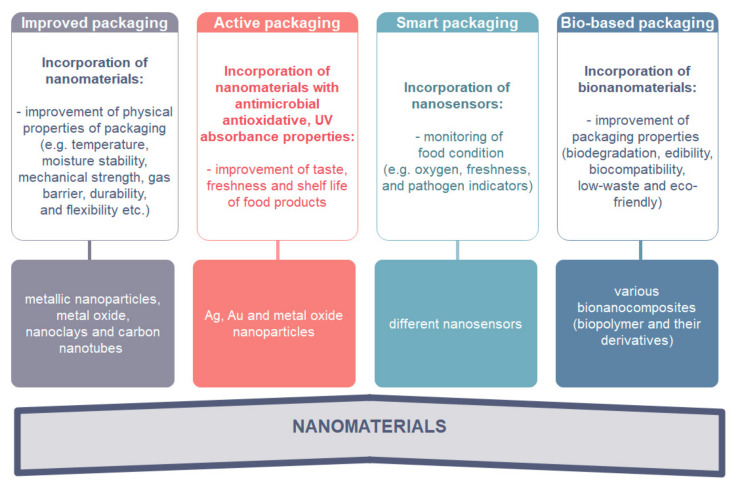
Classification of food packaging based on functional nanomaterials [10].

Food packaging can be improved by usage of functional nanomaterials with physico-chemical improvement, such as temperature and moisture stability, barrier properties, mechanical strength, durability, and flexibility (Improved packaging). The packaging can be also improved using nanomaterials with active functions, e.g., antimicrobial, antioxidative, UV protective etc. (Active packaging), nanosensors with their smart or intelligent functions for the detection of gases and small organic molecules, active stage, and product identification (Smart packaging). Bio-based improvement such as biodegradability, biocompatibility, low-waste and eco-friendly packaging (Bio-based packaging) can also be achieved with incorporation of bionanomaterials [20]. Non degradable plastic polymers which usually serve as actual packaging may be replaced by bio-based packaging, including biodegradable packaging and biocompatible packaging [18]. Improvements of food packaging properties based on functional nanomaterial are presented in Figure 2.

### 2.1. Improved Food Packaging

The basic goal of improved food packaging by incorporation of functional nanomaterials into polymer material is to increase the mechanical and physical properties of the packaging such as gas barrier properties, resistance to temperature and humidity, mechanical strength, and flexibility. Various nanocomposites or nanoparticles incorporated in polymers and nanostructures are already manufactured for the purposes of the various beverages and oils industry, where the proportion of nanoparticles such as clay nanoparticle composites is up to 5% (*w*/*w*). These nanomaterials improve barrier properties of packaging such as reduction in oxygen and carbon dioxide permeation for up to 80–90% [21]. Kim and Cha [22] reported that excess clay loadings in ethylene-vinyl alcohol (EVOH) copolymer-based nanocomposite films lead to the reduction of tensile properties and optical transparency due to the formation of clay agglomerates. With the addition of only 3% (*w*/*w*) clay, the oxygen (by 59%) and water vapor barrier (by 90%) properties of the nanocomposite films were improved in comparison to the material without added montmorillonite clay nanoparticles.

**Figure 3:**
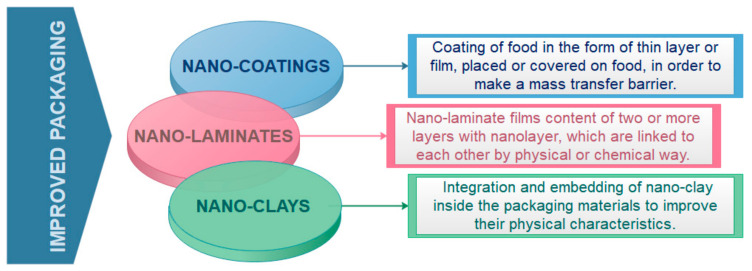
Nanotechnology methods for improvement of mechanical and physical properties of food packaging.

Arora et al. [23] reported that the inclusion of 4% (*w*/*w*) nano clay resulted in an increase in oxygen barrier properties of polystyrene (PS) by 51%. Different nanotechnology methods for improvement of mechanical and physical properties of the packaging are depicted in Figure 3.

**Figure 4:**
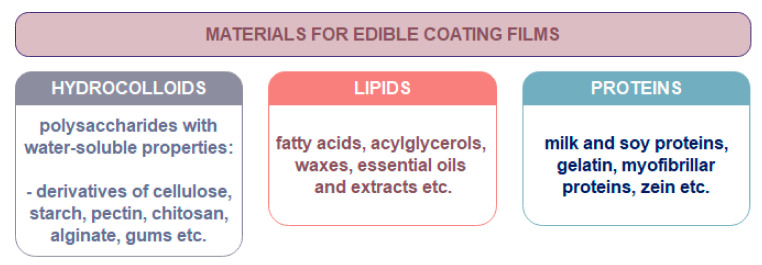
Materials used for production of edible coating films in food industry.

Nanomaterials suitable for food packaging have many advantages over conventional packaging materials. The most widespread nanotechnology method for improving food packaging properties is nano-coating. Different forms of food coating such as thin layer or film can be used to cover the food to provide mass transfer barrier. These coatings can be also from edible material (Figure 4). Edible food coatings are suitable for direct application on the product, while nonedible coatings have a role of protective container with or without being part food product [12]. The application of edible nano-coatings (~5 nm thin coatings) can be found in meat processing industry, agriculture industry (for protection of fruit, vegetables products), cheese and bakery industry etc. to provide flavor, color, enzymes, antioxidants, and anti-browning compounds to the products [24,25]. Additional, nano-clays as nanomaterials are also significantly used and studied for food packaging because of their mechanical, thermal, and barrier properties, and low cost [26]. Various adsorbing substances such as natural polyelectrolytes (proteins, polysaccharides), charged lipids (phospholipids, surfactants), and colloidal particles (micelles, vesicles, droplets) can be used to form different layers for the preparation of nanolaminates for food packaging [27].

The biopolymers for biodegradable film preparation for food packaging should be renewable, cheap and, if possible, produced from wastes. Among the materials for biodegradable coatings films preparation, polysaccharides such as cellulose, chitosan, starch, pectin, alginate, carrageenan, pullulan and kefiran are mostly studied [28]. They can form coating films with good barrier properties against the transport of O_2_ and CO_2_. Their tensile strength values are similar to those observed in synthetic polymers [29]. For example, tensile strength values of films based on high amylose starch are comparable to those values found in low-density polyethylene films [30]. The combination of polysaccharides with other materials to improve the barrier and mechanical properties of coating films for food application are often studied presently. Usually, the addition of plasticizers (hydrophilic to increase and hydrophobic to decrease water vapor permeability) is required in order to obtain protein- and polysaccharides-based films [31]. Plasticizers improve the mechanical properties of films. Addition of plasticizers enables the reduce in tension, hardness, density, and viscosity of materials. Besides, the polymer chain flexibility as well as the resistance to fracture can be improved [32]. Commonly used plasticizers are depicted in Table 1.

Edible nano-coatings are easy to apply by spraying, immersion, or rubbing [33]. They usually consist of environmentally friendly materials and they do not need to be eliminated from food before consumption [34]. Presently, these nano-coating films present nanostructures in which natural ingredients with antimicrobial and antioxidant activity can be incorporated to increase their beneficial effects on the fresh produce quality [35].

Nano-laminate films are usually consisted of two or more layers with nanolayer, whose interlinkage may be physical or chemical. The most common technique for their synthesis is layer by layer deposition. This method enables surface lamination with multiple nano-layers as interfacial films based on various nanomaterials [36]. Different adsorbing compounds such as natural or biobased polyelectrolytes (polysaccharides, proteins), colloidal particles (vesicles, micelles, droplets), and charged lipids (phospholipids, surfactants) could be used to provide and improve different layers properties [10]. Additional, different active compounds (e.g., antimicrobials, antioxidants, anti-browning agents, enzymes, flavors, odor etc.) can be incorporated into the films [37] with the aim to extend the quality and shelf life of packaged food products [38]. Nano-laminated coatings could be also prepared from edible or biobased ingredients as edible nano-coated films (See Figure 4).

**Figure 5:**
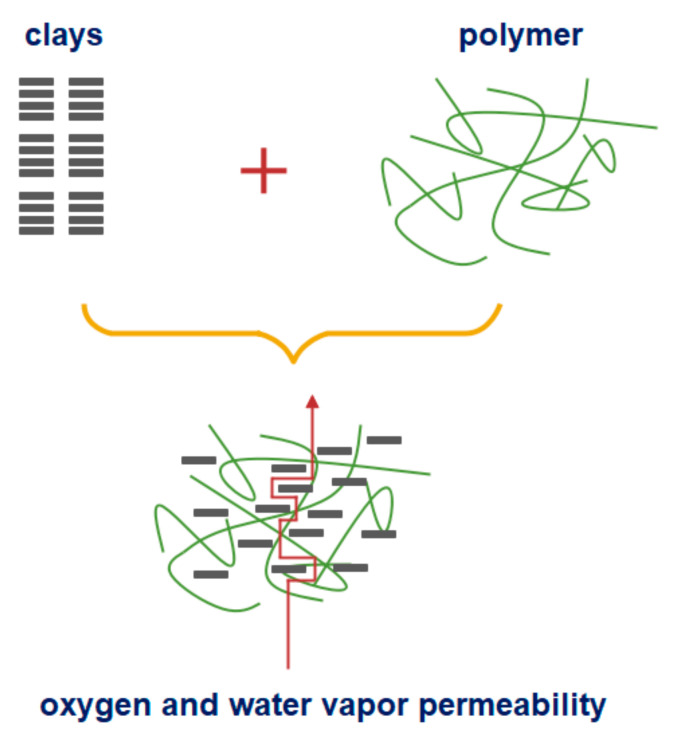
Nonlinear and prolonged pathway of oxygen and water vapor permeability formed due to the incorporation of clay into a polymer matrix film [40].

Polymer/clay nanocomposites also represent the promising classes in food packaging due to the significant increase in the mechanical and barrier properties of these packaging owing to the synergism between the matrix polymer and nano clay, as a small amount of nanofiller is incorporated in the polymer matrix [39]. Food packaging materials based on clay nanocomposite provide enhanced shelf life, lightweight, heat resistant and are shatterproof. In a nanocomposite with included clays, transport of diffusing molecules is obstructed due to impenetrable particles/clays. Consequently, interfacial zones that have different permeability characteristics than those of the basic polymer are formed (Figure 5). Consequently, the nonlinear pathway increases the mean gas diffusion length, which contributes to the prolongation of shelf life of quick spoiled foods.

Presently, research is focused on the use of biocomposites reinforced with natural fibers whose advantage depends on the behavior and properties of natural fibers as reinforcing fillers [41]. The most interesting aspect of natural fibers is their positive impact on the environment [42]. The poor interface quality between the fiber and matrix polymer leads to the poor stress-transfer efficiencies and water absorption properties of natural fibers, thus represent also a negative aspect for the possible industrial application of these materials in the food industry [43]. In Table 2 some examples of different nanotechnology methods for preparation of nanomaterials with improved properties are presented.

### 2.2. Active Packaging

Third of food produced for human consumption (approx. 1.3 billion tons/year) is discarded globally every year. As a major contributor, food spoilage represents a serious environmental problem [61] and consequently affect human health. Besides, it leads in a large economic deficit and raising medical care expenses. Therefore, the development of new technologies and materials for food waste reduction and for improvement of food safety is required. One of the possible strategies to reduce spoilage of food and related increasement in food waste is development of active materials for active packaging to extend the product shelf life. Traditional materials for food packaging are made from non-degradable polyethylene (PE), polypropylene (PP) and polyethylene terephthalate (PET), where molecules penetration of O_2_ and H_2_O is prevented.

Presently, new materials with improved functional properties enable prolonged shelf life of food products. New active food packaging includes different scavengers, absorbers, emitters, coatings (Table 3). These agents could be included in conventional non-degradable packaging, but more and more often they are used in conjunction with biodegradable components.

Additional, emitting sachets or coatings containing antimicrobial agents, antioxidants, flavors, and preservatives with the aim to improve food quality and safety are often part of new generation active packaging.

#### Antimicrobial Active Packaging

The purpose of antimicrobial packaging usage is preservation of foods and extending their shelf life by inhibiting the microorganism’s growth. This could be achieved either by incorporation of an active agent onto or applying a coating layer within the packaging material [61]. Due to different physiologies, antimicrobial agents act differently depending on the pathogenic microorganism.

**Figure 6:**
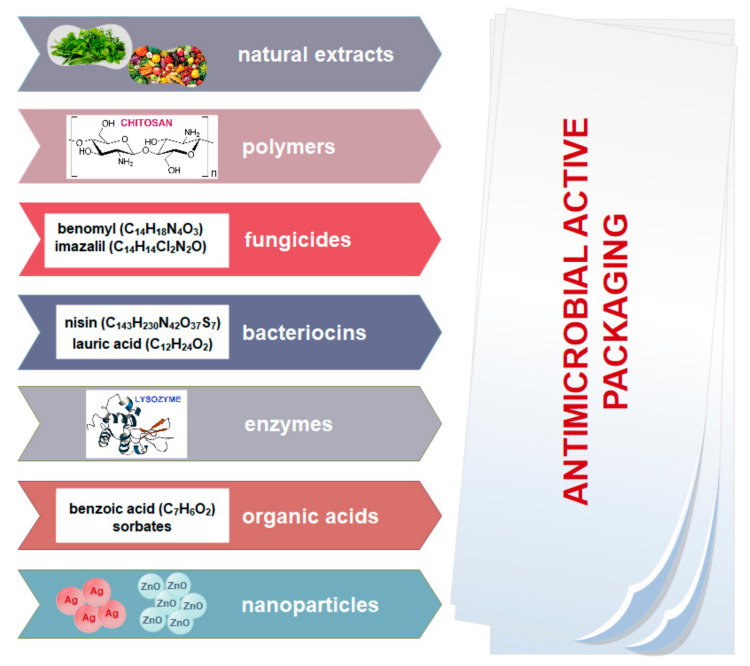
Different antimicrobial substances for antimicrobial active packaging application.

Characterization of microorganisms such as cell wall composition (Gram-negative or Gram-positive), oxygen requirements (aerobes or anaerobes), growth stage (spores or/and vegetative cells), acid/osmosis resistance, optimal growth temperatures (mesophilic, thermophilic …) is basic criterion for the choice of proper antimicrobial agent [68]. Different microorganisms such as *Salmonella spp.*, *Staphylococcus aureus*, *Listeria monocytogenes*, *Bacillus cereus*, *Escherichia coli* O157:H7, *Pseudomonas, Klebsiella*, *Lactobacillus spp*. (spoilage microorganisms bacteria); *Rhizopus*, *Aspergillus* (molds); and *Torulopsis*, *Candida* (yeasts) are responsible for food spoilage [67]. There are two mechanisms of antimicrobial agent action: inhibition of the essential metabolic pathways of microorganisms (e.g., EDTA and lactoferin as coupling agents of charged polymers) or destruction of cell wall/membrane structure (e.g., lysozyme). Various antimicrobial substances with the possibility of incorporation into food packaging systems are presented in Figure 6.

Nanomaterials are often used to improve the properties of food packaging due to their antimicrobial, UV protection activity, and possibility of oxidation prevention, etc. Antimicrobial nanomaterials such as Ag, TiO_2_, ZnO, magnesium oxide (MgO) etc. nanoparticles are due to their high antimicrobial activity very suitable agents for antimicrobial active packaging systems [69]. TiO_2_ nanoparticles as non-toxic to human body and approved as a food additive and for food contact material are often applied to food packaging [70]. Further studies on the post digestion and adsorption effect to the body are required to ensure its safe use in the food industry [71].

Metal-based nanoparticles, as active agents, are often used in the combination with other antimicrobial agents and in the combination with other various metal nanoparticles [72,73,74]. Nanoencapsulation techniques can be also used for entrapment of essential oils with the aim of their stabilization during processing and to improve their physicochemical properties as well as to enhance their health-promoting effects. Herbs and spices present potential high valuable sources of renewable and biodegradable chemicals, such as polyphenols, which show high antioxidant/antimicrobial properties. Therefore, they are desirable substances for incorporation in active food packaging [75]. In particular, essential oil-loaded biopolymeric nanocarriers show promising antimicrobial and antioxidant activity and are suitable material for active food packaging due to inhibition of microbial growth in different food products [76]. Plasticized polylactide (PLA) with loaded bimetallic silver-copper (Ag-Cu) nanoparticles and cinnamon essential oil (CEO) film was used for packaging of chicken meat. The composite films for chicken samples packaging were tested against *Salmonella Typhimurium*, *Campylobacter jejuni* and *L. monocytogenes*. Active packaging film with Ag-Cu nanoparticles and 50% CEO showed maximum antibacterial action over 21 days when the samples were stored in the refrigerator [77]. Buckwheat starch (BS) films containing ZnO nanoparticles showed very good antimicrobial activity, when the concentration of ZnO nanoparticles was 3%, since the obtained film for fresh-cut mushroom packaging demonstrated antimicrobial activity against *L. monocytogenes*, since the reduction of 0.86 log CFU/g after 6 days of storage was detected [78]. Maximum CFU reductions of 96% and 64.1% on *E. coli* growth were obtained using 50/50 ratio of TiO_2_/ZnO nanoparticles-coated low-density polyethylene (LDPE) films at the presence of UV light for film alone and fresh calf minced meat packed, respectively [79]. Montmorillonite clay and ginger extract mediated Ag nanoparticles were used to prepare antibacterial polyvinyl alcohol-based nanocomposite with improved film properties. The nanocomposite clay blend film which had in situ generated Ag nanoparticles, showed clear antimicrobial activity against both *S. Typhimurium* (Gram-negative bacteria) and *S. aureus* (Gram-positive bacteria), with significant action in the case of *S. Typhimurium* [80]. Different polyethylene films containing Ag, clay, and TiO_2_ nanoparticles were produced as a potential active packaging film with the aim to improve fresh chicken shelf life stored at 4 °C. The results demonstrated that a film containing 5% Ag and 5% TiO_2_ nanoparticles had the greatest antimicrobial effect on gram-positive (*S. aureus*) and gram-negative bacteria (*E. coli*) [81]. Polylactide/poly(ε-caprolactone)/ZnO/clove essential oil (PLA/PEG/PCL/ZnO/CEO) composite antimicrobial films were successfully used for scrambled egg packaging with high antibacterial activity for 21 days storage at 4 °C against *S. aureus* and *E. coli*. The complete inhibition of *E. coli* was found for the PLA/PEG/PCL/ZnO/CEO film which indicates the synergism between eugenol–the active compound from the clove oil and the ZnO [82]. Nanocomposite poly (ethylene oxide) films functionalized with Ag nanoparticles and *Acca sellowiana* extracts also demonstrated antimicrobial activity against *E. coli* and *S. aureus* [83]. Biodegradable chitosan-whey protein-based film containing 2% TiO_2_ and Zataria multiflora essential oil exhibited strong antimicrobial properties against *S. aureus*, *E. coli*, and *L. monocytogenes* [84]. Gelatin-based nanocomposite with incorporated chitosan nanofiber and ZnO nanoparticles (G/CHINF/ZnONPs) were tested as active packaging for chicken fillet and cheese. High antibacterial activity of tested nanocomposites against foodborne pathogenic bacteria was detected. Neat gelatin film for packaging shows 1.4- fold lower antibacterial activity against *P. aeruginosa* (5.6 ± 0.1 log CFU/g) and *E. coli* (5.6 ± 0.3 log CFU/g) after 12 days of storage in comparison to G/CHINF/ZnONPs packaging. [85]. Tragacanth/hydroxypropyl methylcellulose/beeswax biocomposite film reinforced with Ag nanoparticles for potential use as food active packaging was tested against pathogen bacteria. The results show that the prepared biocomposite film inhibited the growth of gram-positive pathogen (*B*. *cereus*, *S*. *aureus*, *Streptococcus pneumoniae* and L. *monocytogenes*) and gram-negative (*E*. *coli*, *S*. *typhimorum*, *P*. *aeruginosa* and *Klebsiella pneumoniae*) in a dose-dependent manner. The addition of a higher amount of AgNPs to the biopolymer matrix resulted in higher inhibition growth of bacteria [86]. Additional, polyethylene coated with chitosan-ZnO nanocomposite films shows very high antimicrobial activity. Completely inactivation and additional prevention of food pathogens growth such as *Salmonella enterica*, *E. coli* and *S. aureus* after 24-h incubation was observed [87]. Antimicrobial activity of polyvinyl alcohol-chitosan films with incorporated biogenic Ag nanoparticles showed bactericidal effects against *E. coli* O157:H7, *L. monocytogenes*, *S. aureus*, and *B. cereus* with >99.9% reduction [88]. Antimicrobial properties of biodegradable film based on wheat gluten/ZnO nanocomposites (WG/ZnO) for the active packaging of food products were investigated. Pure gluten film possessed no antimicrobial activity against *E. coli* and *Aspergillus niger*, but films with incorporated ZnO nanoparticles showed a significant antimicrobial activity against bacteria and fungi [89]. Promising antibacterial activity of film composed with cellulose nano whisker (0.5%)-sodium alginate (3%)-copper oxide nanoparticles (5 mM) against various pathogens in terms of higher zone of inhibition against *S. aureus* (27.49 mm), *E. coli* (12.12 mm), *Salmonella sp.* (25.21 mm), *C. albicans* (23.35 mm) and *Trichodenna spp.* (5.31 mm) was detected [90]. Additional, essential oil of clove buds was extracted and encapsulated in chitosan nanoparticles, which possess high antibacterial activity against *L. monocytogenes* and *S. aureus* [91]. Cellulose nanofibrils-soybean oil composite with excellent flexibility, optical transparency, thermal stability, and biodegradable properties films were developed. Nanocomposite film with encapsulated curcumin were prepared to obtain nanocomposite with antioxidant (radical scavenging) properties and antibacterial activity against *E. coli* [92]. Nanocomposite film of chitosan with incorporated TiO_2_ nano-powder showed improved mechanical properties and a very high antimicrobial activity (100% reduction in growth after 12 h) against pathogenic fungi *C. albicans* and *A. niger* and bacteria *E. coli* and *S. aureus* [93].

The presented studies demonstrated that metal/metal oxide nanoparticles as active agents included in the polymer matrix showed a broad antimicrobial activity against pathogenic microorganisms accountable for microbial quality of food product limitation. Furthermore, using such active packaging, the amount of preservatives commonly added directly into the bulk of food can be reduced [74,94]. Active packaging technology passively protects food items, since inhibits the pathogenic growth, provides an extended shelf life combating a variety of environmental factors [68]. Higher surface area-to-volume ratio of nano material antimicrobial agents in comparison to classical material, enables their efficient inhibitory activity against food pathogens. Additional, active food packaging with incorporated antimicrobial agents have shown also enhanced thermal, physicochemical, mechanical, and optical properties [95,96,97,98].

The great antimicrobial and antioxidant potential of enzymes makes them prone to be used as agents for active packaging materials. The major drawbacks of easy usage of native enzymes for active packaging preparation is in the reduction of protein stability when they are in solution. Immobilization of enzymes on solid supports to create biocatalytic interfaces has instead been proven to increase their stability and efficiency [99,100,101,102,103,104]. A recombinant fungal laccase isoform was immobilized into hydrogel films and was tested as potential antitoxin (aflatoxin M_1_ and B_1_) agent for food packaging. The overall catalytic efficiency of the new antimicrobial biofilm increased by a factor two compared to the pure enzyme dissolved in solution with 60-times lower amount of lysozyme needed to achieve a comparable antimicrobial activity [105]. Lysozyme was immobilized onto nanocellulose as a potential nano-biopolymer for food packaging application. Lysozyme-conjugated nanocellulose (LCNC) possessed antifungal and antibacterial effects against *C. albicans*, *A. niger*, *S. aureus*, and *E. coli* [106]. The antimicrobial proteins lysozyme and lactoferrin were incorporated into paper containing carboxymethyl cellulose for thin cuts of raw meat packaging. The prepared food packaging paper exhibited good antimicrobial effect against *Listeria* [107]. β-Galactosidase from *Aspergillus oryzae* was electrospuned into polyethylene oxide (PEO) nanofibers with polyethylene oxide/polypropylene oxide block copolymer (Pluronic F-127) to enable dry storage stability for bioactive packaging [108].

### 2.3. Smart Packaging

**Figure 7:**
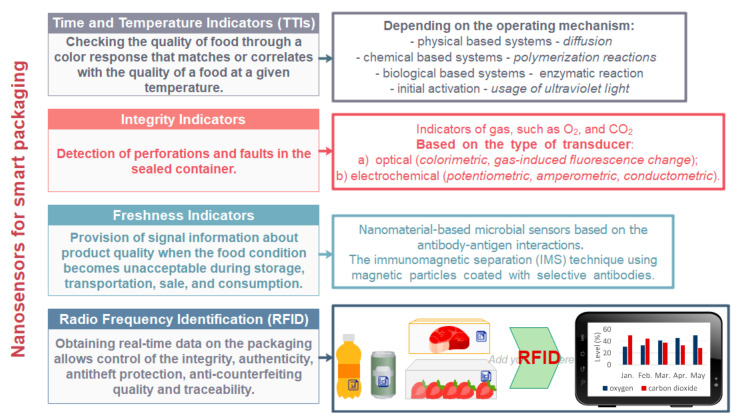
Nanosensors used in smart packaging for food applications [111,112].

The improvement of food packaging with smart or intelligent functions can be performed using nanoparticles to monitor chemical or biochemical, or even microbial development inside the food and or the environment surrounding the product. Therefore, specific pathogens and specific gases nanosensors can be used for food spoilage detection [18,109]. Due to unique optical properties and high surface reactivity of nanomaterials such as metal nanoparticles or photonic nanocrystals, their superior performance in comparison to the traditional colorimetric indicator can be detected. The application of optical indicator, among nanosensors, is often present in the commercial market due to the convenient and easy use [110]. Using nanosensors, the response to internal or external parameter changes inside the food and/or in his surrounding environment is performed, with feedback information to the customer, to ensure food quality and safety. Therefore, different nanomaterials are used with the aim to improve and upgrade the functionality of packaging (Figure 7).

Nanosensors have great potential to fast detection, identification, and quantification of pathogen microorganisms, decaying substances, and allergy-causing proteins E Custom-made nanosensors used in smart packaging are used for food analysis (detecting toxins, chemicals, and food pathogens) detection of flavors or colors etc. [113]. Food packaging can be equipped by nanosensors which are sensitive to humidity, gases formation, or temperature changes and for example when gas is formed due to spoilage of food, the packaging change color of the indicator and thus alerting the customer to the unsuitability of the product. Food packaging equipped with nanosensors, can be successfully used for real-time monitoring of food freshness status, and reduce the requirement for determining the shelf life (expiry date) of the food, since the nanosensors can respond to certain chemical markers, pathogens, and toxins in food [114,115]. A biosensor is device with incorporated biological sensing element connected to a transducer. The analyte that this sensor detects, and measures may be purely chemical (even inorganic), although biological components maybe the target analyte. The key difference is that the recognition element is biological in nature [116]. Bionanosensors are the result of combining the biosensors and nanotechnology. In Table 4 different applications of bionanosensors in the field of food packaging are presented.

Additional, nanosensors can be used also for sensing the quality of food ingredients as detector for pesticides and chemicals [139,140,141,142,143,144,145,146], unstable food ingredients [147,148,149,150] etc.

### 2.4. Bio-Based Packaging

Another type of packaging is bio-based packaging or biochemical improved packaging, which is more and more often applied in the field of food packaging. Therefore, the market of some bio-based and/or biodegradable plastics such as bio-poly(ethylene terephthalate), polybutylene succinate, PLA are expected to grow significantly in the following years [151]. Global bioplastics production capacity is projected to increase sharply in the coming years for more than 15%; from around 2.11 million tonnes in 2019 to around 2.43 million tonnes in 2024 [151]. Bio-based packaging materials are more environmentally friendly compared to traditional plastic packaging. Besides, bio-materials provide protection between food and the surrounded environment, thus avoiding the deterioration of food quality, such as decontamination with microorganisms, changes in gas conditions, and the relative humidity of the environment and especial they can be degradable using living microorganisms [152,153,154].

**Figure 8:**
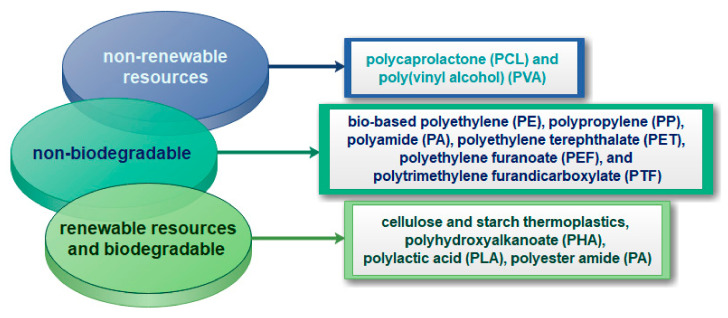
Bio-based materials for food packaging applications [151,155,156].

In general, these materials are environmentally friendly and can be completely degraded into CO_2_, water, and biomass. Bio-based packaging nanomaterial are produced from renewable resources, consequently energy can be saved by incineration of bio-based materials [10]. Various routes have been developed in the past decade to produce bio-based materials [151]. The bio-based materials can be made from renewable biomass or non-renewable resources and are biodegradable or non-biodegradable (Figure 8).

The biodegradable polymers could be categorized based on their origin: (a) polymers, obtained from biomass, (polysaccharides, polynucleotides, polypeptides, proteins etc.), (b) polymers, synthesized from bio-monomers or obtained from mixed biomass and petrochemicals, (polylactic acid or bio-polyester), (c) polymers, produced by microorganisms (bacterial cellulose, polyhydroxybutyrate, xanthan, etc.) [10]. The problems associated with biodegradable polymers is that the bio-based and biodegradable polymers are currently more expensive than fossil-based polymers [157]. However, due to specific material properties, the reduction in final cost also considering the end-of-life phase of material can be expected. As production scale, final product conversion and logistics become more favorable, bio-based polymer material prices are expected to decline. Additionally, the use of nanotechnology in preparation/modification of bio-polymers could create the new opportunity for improving the physical and chemical characteristics of biopolymers and decreasing in price effect. Presently, the common bio-nanocomposites used for food packaging material are starch and its derivates, such as PLA, polyhydroxybutyrate (PHB), polybutylene succinate (PBS), and aliphatic polyester, chitosan, proteins, and cellulose. Therefore, bio-nanocomposites polymer could be used besides for biochemical improved packaging, also for improved packaging containing active functions. Hence, bio-nanocomposites polymer could be employed not only for biochemical improved packaging but also for improved packaging with active functions.

#### 2.4.1. Starch-Based Nanomaterial

Starch is cheap, widely available, film forming, renewable, and biodegradable. Therefore it is a very interesting and promising biopolymer for food packaging application, but possess weak barrier properties, water sensitivity, and brittleness when it appears in native form [158]. The use of starch material in combination with other materials to reduce the weaknesses of this natural polymer has led to an increase in its usage in various industries, especially in packaging industry [159]. The improvement of their mechanical, UV and water barrier properties can be obtained also by incorporation of different nanoparticles such as TiO_2_, ZnO nanoparticles, graphene, and poly(methyl methacrylate-co-acrylamide) in the polymer structure [160,161,162,163]. The effect of graphene oxide with different concentrations in the performance of the starch/PVA/graphene oxide composite film was studied. The addition of graphene oxide (optimal concentration 2 mg/mL) can improve the mechanical properties, transmittance, and water vapor permeability of the composite film [164]. Antimicrobial nanopackaging films against *E. coli* and *S. aureus* were developed by incorporating clove essential oil (15–30% (*w*/*w*)) and graphene oxide nanosheets (1% (*w*/*w*)) into PLA, where optical and anti-UV properties of the film were influenced by incorporation of graphene oxide nanosheets and essential oil [165].

Starch nanoparticles (SNPs) or nanocrystals are due to their unique functional properties suitable for food application, including for biodegradable food packaging. They are obtained from starch after acid or enzymatic hydrolysis or other mechanical methods [166]. Pea starch nanocrystals dispersion containing nanocrystals in a range of 30–80 nm was used for the preparation of films by blending PVA with native pea starch and pea starch nanocrystals, respectively. The PVA/pea starch nanocrystals nanocomposite films containing 5 and 10% (*w*/*w*) of nanocrystals exhibited improved physical properties over the PVA film [167]. Corn starch films were prepared using taro starch nanoparticles (TSNPs), which were obtained by hydrolysis with pullulanase and the recrystallization of gelatinized starch. With the incorporation of TSNPs to the corn starch films, the decrease in vapor permeability and increase in opacity was detected. Additionally, the highest tensile strength (2.87 MPa) was detected when the starch films contained 10% (*w*/*w*) of TSNPs and also improvement in thermal stability of starch films was observed [168]. For the potential use as food packaging membrane application, composite membrane composed from biodegradable poly(ε-caprolactone) (PCL) and platelet-like starch nanocrystal (SNC) particles was prepared. The presence of 1% (*w*/*w*) SNC particles in membrane improved gas barrier properties (reduction in oxygen transmission rate by about 70%), tearing strength (increasement by about 68%), and creep resistance of the PCL membrane [169]. The rice starch-based film reinforced with starch nanocrystals showed an increase in tensile strength but decreasing elongation at break and water barrier properties with the addition of rice starch nanocrystals. Besides, the addition of lower content of starch nanocrystals increased the crystalline peak structure of rice starch film [46]. Citric acid-modified starch nanoparticles with an average size of 82 nm were incorporated in glycerol-plasticized soy protein plastics. Due to incorporation of modified starch nanoparticles, improvement in tensile strength and Young’s modulus while a slight decrease in elongation at break was observed. Besides, the water uptake decreased when the nanoparticles were presented in polymer [170]. Nanocomposite films were prepared via the casting of a mixture of latex dispersions of poly(butylmethacrylate) with the nanoparticles or nanocrystals from waxy maize starch. The starch nanocrystals were more suitable for reinforcing a polymer film, since 2.5× modulus improvement at 10% (*w*/*w*) nanoparticles loading when compared to starch nanoparticles was achieved. While the reduction in transparency was less intensive when the nanoparticles were used in comparison to nanocrystals due to their smaller size [171].

#### 2.4.2. Cellulose-Based Nanomaterial

Cellulose, obtained from lignocellulosic biomass, became popular due to its ecological and biodegradable nature. The great potential applications in the area of food packaging are attributed to different types of nanocellulose such as cellulose nanofibrils, cellulose nanocrystals, bacterial nanocellulose, and nanocellulose-based hybrid nanomaterials [172]. Cellulose nanofibers, eco -friendly nanomaterial, have great potential in food packaging also from the economical point of view due to decrease in costs and reduction of environmental impact [173]. Cellulose nanoparticles are often used for reinforcement of polymer composites for the food packaging applications. Various materials, as a matrix, such as alginate [174], chitosan [175], PLA [176], polycaprolactone [177], glucomannan [178], pectin [178] etc. have been incorporated with cellulose nanoparticles by various techniques. The nanoscale structure and the high specific surface area of cellulose enables cellulosic nanocomposites remarkable mechanical, biodegradation, optical and barrier properties. The cellulose nanoparticles improve mechanical properties of polymer composites at lower added amount, opposite to higher number of added nanoparticles which induces agglomeration, resulting in poor mechanical properties (Table 5).

The packaging films have been made from bacterial cellulose, produced from cashew apple juice, with addition of lignin (up to 15% (*w*/*w*)) and cellulose nanocrystals (up to 8% (*w*/*w*)), both obtained from waste cashew tree pruning fibers. Produced films possessed enhanced tensile properties and decreased water vapor permeability. Furthermore, the films had improved UV-absorbing and antioxidant properties, making them interesting for food products packaging sensitive to lipid oxidation [180]. Additional, cellulose nanocrystals (CNCs) incorporated with supermagnetic iron oxide nanoparticles (Fe_3_O_4_ NPs) were used for the preparation of high-performance nanocomposite films consisting of poly(3-hydroxybutyrate-co-3-hydroxyvalerate (PHBV) and nanocrystal-nanoparticle hybrids (MCNC). When the CNCs were incorporated in MCNC-5% hybrids, the surface hydrophilicity of PHBV was improved, and water vapor permeability was reduced by 68% in comparison to basic PHBV. Besides, MCNC hybrids can have a function of sensor for voltage change detection of the nanocomposites as operative voltage response signal with real-time monitoring of water vapor [181]. Soy protein isolate (SPI)-based active nanocomposite films with incorporated cellulose nanocrystals (CNC) and zinc oxide nanoparticles (ZnONP) were prepared by two different method: as CNC/ZnONP mixture (physical mixing), or CNC@ZnONP nanohybrids (in situ growth). Particularly due to incorporation of CNC improved in tensile strength, oxygen and water vapor barrier properties, water resistance ability, and thermal stability were determined. In addition, the synthesized film with ZnO inhibited the growth of foodborne pathogens (*E. coli* and *S. aureus*) and were used for reduction of foodborne pathogens and the total volatile basic nitrogen values in a pork sample [182]. The antimicrobial efficacy of the gelatin-based nanocomposite films containing cellulose nanofibers (CNF) and oxide nanoparticles (G/CNF/ZnO NPs) as a food packaging material, was tested again *S. aureus* and Pseudomonas fluorescens inoculated on chicken fillets. The usage of antibacterial film caused a significant reduction in population of bacteria on the chicken fillets, especially against *S. aureus*. Mechanical properties of nanocomposite film containing 5% CNFs were significantly improved; Young’s module by 47%, tensile strength by 72%, but decreased in flexibility (by 28%), water vapor permeability, and moisture absorption was observed [183]. Today, concerns about the potential toxicity associated with the use of nanoparticles in foods expressed by consumers, regulatory agencies, and the food industry are at a high level. Therefore, studies performed in this area are highly recommended. The toxicity of PVA-based films with incorporated cellulose nanofibril/TiO_2_ nanoparticle nanocomposites was tested against Bif-6 cells. Nanocomposites did not show significant toxicity to cancerous and normal colon cells, regardless of the increasement of their concentration to 1000 µg/mL [184].

#### 2.4.3. Chitosan-Based Nanomaterial

**Figure 9:**
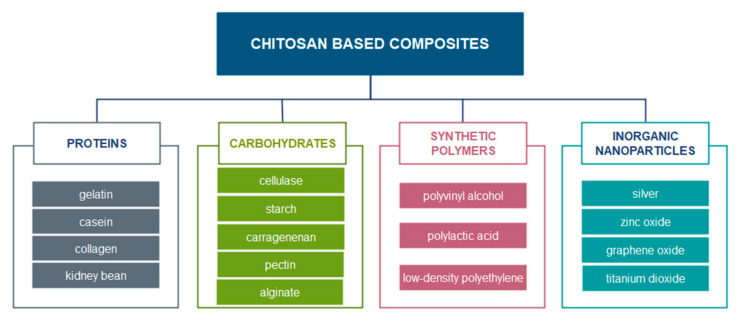
Materials for chitosan-derived composites processing for food packaging application [192].

Chitosan is one of the most studied polysaccharides and is the second most abundant polysaccharide in the world which can be obtained from plentiful renewable sources. Therefore, it is inexpensive and commercially available [185]. However, the main drawbacks of chitosan usage as packaging material in comparison to the generally used non-biodegradable polymers from petroleum are reflected in its poorer mechanical, thermal and barrier properties [186]. With usage of nanotechnology improvement in functional properties of chitosan matrix by the incorporation of newer nanoparticles, other polymers, and components (Figure 9) can be achieved. Therefore, presently, chitosan films are increasingly used as packaging materials to maintain the quality of preserved foods. Chitosan has exhibited high antimicrobial activity against a wide variety of pathogenic and spoilage microorganisms, including fungi, and Gram-positive and Gram-negative bacteria [185,187,188]. Generally, lower molecular weight chitosans (of less than 10 kDa) have greater antimicrobial activity than native chitosan. A degree of polymerization of at least seven is required, highly deacetylated chitosans are more antimicrobial than those with a higher proportion of acetylated amino groups, due to increased solubility and higher charge density [189]. There are two most probably mechanisms of chitosan antimicrobial action: (a) chitosan binding to the negatively charged bacterial cell wall, which leads to disruption of the cell and therefore the membrane permeability is altered, so inhibition of DNA replication is leading to cell death [190]; or (b) action of chitosan as a chelating agent that can be bound to trace metal elements and consequently, the toxins are formed resulting in inhibition of microbial growth [191].

Mechanical and barrier properties of chitosan-based material are depended on the type of chitosan, but general the chitosan-based films are suitable for food packaging and active packaging. The main drawback for the application of chitosan solutions for formation of film is the acid pH. With the increase in pH of the solution above 6.2, the formation of a precipitated hydrated gel occurs and the reason is neutralization of the amino groups [185]. For further improvement in antimicrobial efficacy and other mechanical properties of natural polymer, incorporation of other molecules into chitosan-based polymer is proposed to develop new composites with improved properties applicable for food packaging [193]. Addition of TiO_2_-Ag in fish gelatin and chitosan composite film significantly increased the water solubility of the film. At the concentration of TiO_2_-Ag 0.5%, the highest antibacterial efficacy and lowest light transmittance of 54.6% was detected while under the given conditions a decline in tensile strength was observed [194]. As a promising food packaging material, hybrid PLA/cellulose nanofibers/organically modified nano clay (C30B) composite with lower water vapor sorption and diffusion in comparison to PLA material was prepared by Trifol and coworkers [195]. The incorporation of ZnO and gallic acid into chitosan films have remarkably improved the mechanical and physical properties of composite such as oxygen and water vapor permeability, swelling, water solubility and UV-vis light transmittance. Additionally, the modified chitosan-based composite possessed significant antibacterial and antioxidant ctivity compared to basic chitosan [196]. Polyethylene (PE) and polypropylene (PP) two-layer functional coated films were developed for the active packaging using macromolecular chitosan solution for first, while the second (upper) layer contained catechin and pomegranate extracts (polyphenols) incorporated in chitosan nanoparticles with the aim to provide an antioxidant and antimicrobial properties of food packaging [197]. Ag nanoparticles/chitosan/PVA electrospun fibrous composite nano-layers were successfully used as packaging material for meat with improved bio-activity and extended meat shelf life [198]. Electrospun chitosan-based nanofibers for fresh meat packaging were tested against *E. coli*, *S. enterica* serovar Typhimurium, *S. aureus* and Listeria innocua. The bacterial susceptibility was strain-dependent and non-virulent bacteria showed higher susceptibility (99.9% reduction rate). Also, the prepared nanofibers for food packaging were enabled extending the shelf life of meat product for one week [199]. High, medium, and low molecular weight of chitosan were used as raw materials to prepare a series of films with addition of glycerol for strawberry preservation. The chitosan-based film with high molecular weight, high chitosan content and 50% glycerol/chitosan (*w*/*w*) possessed the excellent transparency (higher than 91%), elongation at break (approx. 29%), tensile strength (approx. 78 MPa), water vapor permeability (1.5 × 10^−12^ gcm/cm^2^.sPa), smooth morphology, denser structure and antibacterial efficiency against *S. aureus* and *E. coli* [200]. Better mechanical and physical properties of high molecular weight chitosan was observed also by Tanpichai and coworkers [201]. Chitosan was also used as coating dispersion for extension of pomegranate arils shelf life during storage at 5 °C. Different dispersions included chitosan, clove essential oil, chitosan nanoparticles, and clove essential oil-loaded chitosan nanoparticles. Among tested dispersions, clove essential oil-loaded chitosan nanoparticles extended aril shelf life for 54 days while uncoated arils were contaminated with fungi after the 18th day of storage [202]. Incorporation of ZnO nanoparticles and linseed oil in chitosan/potato protein-based polymer was used for determination of storage quality of raw meat. Results indicated that the incorporation of ZnO nanoparticles improved the transparency and tensile strength of the films and addition of linseed oil the elastic property of composite film. Furthermore, biopolymer films possessed an excellent moisture barrier capability [203]. Also an improvement in antioxidant, antibacterial and sensory properties was determined when the chitosan-gelatin bio-based edible coating incorporated with nano-encapsulated tarragon essential oils was used to preserve pork slices [204]. Besides, development of active food packaging via incorporation of biopolymeric nanocarriers such as chitosan nanoparticles containing different essential oils (cinnamomum [205], assai pulp [206], thyme [207], lemon [208], Zataria multiflora [84], tarragon [204]) showed oxidation and microbial growth reductions in different food products [76]. The combination of natural polymer and ZnO nanoparticles showed a good synergistic effect on inhibition of foodborne pathogens growth in food application. Al-Nabulsi and coworkers [209] prepared chitosan coating with ZnO nanoparticles (concentration of 0.0125%, which significantly reduced the initial numbers of E. coli O157:H7 in white brined cheese by 2.5 CFU/g, when stored at 4 °C or by 1.9 CFU/g when stored at 10 °C.

## 3. Safety and Environmental Concerns of (Bio)Nanotechnology Implementation in Food Packaging

Nanotechnology is a rapidly developing field and nanomaterials are of significant technological and economic interest and have a huge impact on many industries especially in the food packaging industry. In general, the beneficial effects of nanocomposite materials are well recognized as opposed to the potential (eco)toxicological effects and effects of nanoparticles on human health, where less studies were performed. Their interaction with food system raises a concern about human and animal health. The use of nanomaterials in nanosensing or food packaging applications can lead to the migration of nanomaterials in the human organism. This can occur through inhalation, skin penetration or ingestion due to leaching of nanocomponents from packaging or sensing elements into the food, or by storing of packaging, nanosensors in landfills with possibility of release into the environment, air, water, and soil [210,211,212]. Some performed studies have shown the possible toxicological effects of nanoparticles on biological systems [213,214,215]. However, the toxicity seems to depend on the type and size of the nanoparticles [216]. Side effects caused by nanoparticles exposure include the formation of reactive oxygen species (ROS), protein denaturation, mitochondrial disorder, and phagocytosis dysfunction [70]. A potential concern should be focused on the migration of nanomaterials from the packaging or the sensing element inside the packaging into the food. The most evident contact route of nanomaterials to the human body in food application is upon ingestion. Therefore, the gastrointestinal tract properties such as pH, presence of various surface-active molecules, electrolytes, digestive enzymes, gut microbiota, and mechanical forces influence the absorption of nanomaterials, which may cause changes in the properties and agglomeration state of nanoparticles [217]. The Ag migration from the various kinds of nanocomposites (plastic food containers) into food has been investigated by Echegoyer and coworkers with conclusion that in acidic food the highest level of Ag migration was detected. Besides, microwave heating increased more migration of Ag nanoparticles than a conventional oven [218]. Metal and metal oxide nanoparticles are often specified as biocompatible materials, without significant toxic effect in vivo and in vitro; however, pro-inflammatory responses and oxidative stress due to their presence were also detected [216,219,220]. In addition, the accumulation of carbon nanotube particles in living organisms and the consequent formation of ROS could classify nanotubes as potentially toxic nanomaterials. However, their toxicity is mainly conditioned by its structural modification, size distribution, surface charge and the impurities and functionalization [221,222,223]. Migration of various metal oxides such as TiO_2_, ZnO, SiO_2_, aluminum oxide, which were used in food packaging to improve mechanical, antimicrobial, light-blocking and gas barrier properties of polymers, was investigated. No significant migration of nanomaterials from packaging to food was determined [224], but additional barrier to separate food from nanocomposite is recommended. The possible migration of biopolymers also needs to be studied and taken into consideration. Ubeda and coworkers studied the migration of PLA in the form of pellets and films to food simulants was carried out. Migration tests confirmed the presence of some PLA oligomers in food simulants as well as new neo-formed oligomers formed due to the reaction processes between PLA components and food simulants [225]. Zimmermann et al. made in vitro toxicity tests and chemical composition study of 43 everyday bio-based and/or biodegradable products as well as their precursors, covering mostly food contact materials made of nine material types. 67% of the samples induced baseline toxicity, 42% oxidative stress, 23% antiandrogenicity and one sample estrogenicity. They concluded that bio-based/biodegradable materials with regards to the chemicals they contain and conventional plastics are similarly toxic [226]. However, effective guidelines and policies are required for the safer use of nanoparticles in food industry. The regulation of nanoparticles in food applications and food packaging in the USA is guided by USFDA. Food Standards Australia and New Zealand (FSANZ) is the responsible body for regulation of food additives in nano size and ingredients in Australia. The use of nanoparticles in food applications or food ingredients in EU is regulated by the European Union Novel Foods Regulation (EC 258-97). European Food Safety Authority (EFSA) prepared the re-evaluation program where a scientific opinion on the potential risks on food and feed safety (EFSA 2009), also considering nanoparticles, is drafted. Some existing restrictions and ongoing evaluation processes of nanomaterial safety are already defined (for example existing restrictions of TiO_2_ application in France, chitosan smaller than 100 nm is not allowed in EU for direct food contact applications etc.).

Though, additional research and investigations focused on the physico-chemical characterization, exposure assessment and toxicokinetics and toxicity of nanomaterials are needed to address the many current uncertainties and data limitations of their use in food application. The investigation should study the interaction and stability of nanomaterials in food and feed in the gastro-intestinal tract and in biological tissues. Also, the routine methods to detect, characterize and quantify nanomaterials in food contact materials, food, and feed as well as methodologies to assess toxicity including chronic exposure and carcinogenicity of nanomaterials should be developed [214]. Additionally, internationally granted protocols for the toxicity tests of nanomaterials are required for standardization of data due to their diversity.

## 4. Conclusions and Future Perspectives

The use of nanomaterials is increasing in diverse fields of application drastically. Current research in food packaging suggests that nanotechnology offers a variety of options in the improvement of food packaging based on functionality nanomaterials, from bio-based packaging to smart packaging. Due to the growing demand for types and varieties of exotic foods and the consequent provision of safer packaging of goods, the concept of food packaging will be increasingly advanced in the industry in the future. Nanotechnology, used for the processing of food packaging allows a remarkable improvement in packaging material properties, but further research and development are needed to better understand the role of nanotechnology in the case of food packaging materials, in particular by the advantages and disadvantages of its effect. The usage of nanotechnology in the food sector is focused on improving food quality and safety in form of the incorporation of nanoparticles in food or packaging materials. To create new food packaging functions, the use of nanotechnology enables possible improving the properties of food, such as healthier, tastier as well as improved nutritious food, when it is packaged. Additionally, by employing suitable nanomaterials, the mechanical properties, better barrier, and thermal properties of packaging materials could be improved to prolong food self-life and safety. Such material can be a surface-modified antimicrobial films from nano cellulose with incorporated both inorganic or organic antimicrobial agents with extremely good antibacterial activity against both Gram-positive and Gram-negative bacteria [227]. The nanosensors, as intelligent packaging, could also serve to obtain visual information about the food state inside the packaging. The usage of nanoparticles as a food ingredient is more harmful than their use in food packaging applications. Active, intelligent, and bio-based packaging technologies can work synergistically to create a multi-purpose food packaging system without negative interactions between components, what present future goal of food packaging technology [66]. Many global companies Amcor Ltd., Sonoco Products Company, BASF SE, Tetra Laval International S.A., Honeywell International Inc., and Chevron Phillips Chemical Company, LLC, among others already produce nanotechnology-based packaging materials that extend shelf life and improve food safety. The upcoming trends in the nanotechnology application in food packaging sector are expected to be at the forefront in the coming decade, with dominance in the field of blockchain application [228].

With more intensive development of sustainable or green food packaging, the impact of packaging on the environment could be drastically reduced, through the use of edible or biodegradable materials, plant extracts and bionanomaterials. Besides the human health aspects, the carbon, energy, water, and land footprints need to be taken into consideration when the creation of new food packaging materials is in progress to avoid regrettable substitutions to already existing ones [226].

## Figures and Tables

**Table 1:** Commonly used plasticizers in food application [31,32].

Type	Representative	E Number	ADI Value
polyols	glycerol	E422	not specified
	sorbitol	E420	not specified
	polyethylene glycerol	E1521	0–10 mg/kg body weight
sugars	glucose	-	-
	sucrose	-	-
lipids	monoglycerides	E471	not specified
	phospholipids	(lecithin E322)	not specified
natural source	triglycerides from vegetable oil	(sorbitan tris tearate E492)	0-25 mg/kg body weight
	fatty acid esters	-	-

**Table 2:** Different nanotechnology methods for the preparation of nanomaterials suitable for improved food packaging.

Nano-Technology Method	Composition of Nanomaterial	Function/Properties	Reference
NANO- COATING FILMS	waxy maize starch nanocrystals	Reinforcing agent in a thermoplastic waxy maize starch matrix plasticized with glycerol.	[44]
	starch nanoparticles	Corn starch-based edible films	[45]
	rice starch nanocrystals	Rice starch edible films	[46]
	corn starch/orange-peel oil/zein nanocapsules	Edible films	[47]
	carboxymethyl cellulose/sodium montmorillonite clay/titanium dioxide (TiO_2_)	The addition of NPs decremented water vapor permeability, while moisture content, density, and glass transition temperature were incremented slightly.	[48]
	whey protein isolate/cellulose nanofibers/TiO_2_/rosemary essential oil	Improved physico-mechanical, antibacterial and antioxidant properties.	[49]
	potato starch/sodium montmorillonite clay/TiO_2_	Water vapor permeability and UVA, UVB and UVC lights transmittance decrease upon TiO_2_ and sodium montmorillonite content increase.	[50]
	keratin/polyvinyl alcohol/tris(hydroxymethyl) aminomethane /sodium montmorillonite clay/TiO_2_	Water vapor permeability, oxygen permeability, and light transmittance decrease with increase in TiO_2_ and montmorillonite contents.	[51]
	starch/ polyvinyl alcohol/Ag nanoparticles	High exhibited activity against bacteria and fungi was obtained. Ag release into the non-polar food simulants was lower than into polar simulants.	[52]
NANO- LAMINATES	alginate/chitosan/folic acid	Improved stability under ultraviolet light exposure after folic acid encapsulation.	[53]
	polyethylene terephthalate/aluminum oxide (Al_2_O_3_)/Zinc oxide (ZnO)	Good barrier properties	[54]
	chitosan/alginate/polyethylene terephthalate	Increase in melting energy of 39.2% in comparison to the PET film used as support, and a decrease in the decomposition temperature.	[55]
NANO- CLAYS	clay montmorillonite/pectines	Diffusion of water vapor and oxygen was reduced.	[56]
	chitosan-clay nanocomposites	Addition of clay significantly increased the strength and stiffness of neat chitosan nanocomposite.	[57]
	polypropylene/montmorillonite/ pro-degradant additive (TDPA^®^)	Permeability of oxygen decreased with increasing montmorillonite nano clay content.	[58]
	polycaprolactone/organo nanoclay/chitosan	Antimicrobial effect on *E. coli*, *Pseudomonas aeruginosa*, and *Candida albicans*.	[59]
	corn starch/natural montmorillonite/ anthocyanin	Active and pH-sensitive bionanocomposites with improved mechanical and thermal properties.	[60]

**Table 3:** Functional properties of active food packaging to extend shelf life and improve food safety.

Type	Function	Agents	Reference
oxygen scavengers	prevention of fat oxidation	*metallic* (iron powder, activated iron, Zn …), *organic* (ascorbic acid, tocopherol, catechol …), *inorganic* (sulfite, thiosulfate, ZnO …), *polymer-based* (polymer metallic complex …), *enzyme-based* (glucose oxidase, laccase …)	[62]
ethylene scavengers	fruit and vegetables ripening reduction	SiO_2_, KMnO_4_, TiO_2_, Ag, PdCl_2_, Pd-impregnated zeolite, polyvinyl chloride film containing ZnO nanoparticles …	[63,64]
moisture absorbers	microbial growth reduction	*inorganic* (silica gel, natural clay (montomorillonite, zeolite), chlorides (Ca, Mg, Al, Na, K), oxides (Ca, Ba), bentonite …), *organic* (sorbitol, xylitol, fructose, cellulose and their derivatives), *polymer-based* (starch copolymers, polyvinyl alcohol, absorbent resin)	[65]
carbon dioxide emitters	inhibition of spoilage by microbial action	sodium bicarbonate/ascorbate and citric acid	[66,67]

**Table 4:** Summary of different possible applications of bionanosensors in food packaging.

Application of Bionanosensors	Nanomaterial (Transducer Element)	Bioreceptor	Analyte	Reference
toxins detection	tri-layer oxide (SiO_2_ 10 nm/Si_3_O_4_ 10 nm/SiO_2_ 10 nm)	monoclonal antibodies for aflatoxin-B_1_, zearalenone and HT-2	mycotoxins (aflatoxin-B_1_ and zearalenone)	[117]
	nanopipettes from quartz capillaries	poly l-lysine, polyclonal antibody HPV_16_ E_6_ ad monoclonal antibody for HT-2	HT-2	[118]
	colloid gold nanoparticles	polyclonal antibody for botulinum neurotoxin type B and polyclonal antibody IgG	botulinum neurotoxin type B	[119]
	colloid gold nanoparticles	antibody PbTx Mab and polyclonal antibody IgG	brevetoxins (PbTx-1, PbTx-2, PbTx-3, PbTx-9)	[120]
	carbon nanotubes	antibody of microcystin-LR	microcystin-LR	[121]
	carbon nanotubes	bovine serum albumin, polyclonal anti-palytoxin antibodies	palytoxin	[122]
	gold nanoparticles	cysteamine, monoclonal antibody of aflatoxin B_1_	aflatoxin B_1_	[123]
	carbon dots	aflatoxin B_1_ aptamer HS-AAA AAA GTT GGG CAC GTG TTG TCT CTC TGT GTC TCG TGC CCT TCG CTA GGC CCA CA	aflatoxin B_1_	[124]
	Poly (amidoamine) dendrimers	cysteamine, and aflatoxin B_1_ aptamer NH_2_-5′-GTT GGG CAC GTG TTG TCT CTC TGT GTC TCG TGC CCT TCG CTA GGC CCA CA-3′	aflatoxin B_1_	[125]
microbes detection	single-walled carbon nanotubes	polyclonal antibody for *S. enterica*	*S. enterica* subsp. *enterica* serotype Infantis	[126]
	single-walled carbon nanotubes	ssDNA probes and complementary DNA	*S. enterica* serovar Typhimurium	[127]
	multi-walled carbon nanotubes	*Salmonella* aptamer sequence 5′-T ATG GCG GCG TCA CCC GAC GGG GAC TTG ACA TTA TGA CAG 3′	*S. enterica*	[128]
	single-walled carbon nanotubes	*biotinylated E. coli antibodies*	*E. coli* K-12	[129]
	polypyrrol nanowires	monoclonal antibodies specific toward *Bacillus globigii* spores	*B. globigii*	[130]
	Au nanoparticles	*E. coli* O157:H7-specific antibody, *E. coli* O157:H7 intact cells and *E. coli* O157:H7-specific antibody conjugated with horseradish peroxidase (HRP)	*E. coli O157:H7*	[131]
	Au nanoparticles	*L. monocytogenes* specific antibody	*L. monocytogenes*	[132]
	Fe_3_O_4_ magnetic gold nanoparticles	*S. typhimurium* aptamer sequence 5′-SH-TAT GGC GGC GTC ACC CGA CGG GGA CTT GAC ATT ATG ACA G-3′ and *S. aureus* aptamer sequence 5′-SH-GCA ATG GTA CGG TAC TTC CTC GGC ACG TTC TCA GTA GCG CTC GCT GGT CAT CCC ACA GCT ACG TCA AAA GTG CAC GCT ACT TTG CTA A-3′.	*S. typhimurium* and *S. aureus*	[133]
	carbon dots	amino-modified aptamers of *S. typhimurium*	*S. typhimurium*	[134]
	super-paramagnetic iron oxide particles	monoclonal antibody _12_F_6_ against *Bacillus anthracis*	*B. anthracis spores*	[135]
	magnetic nanoparticles	*E. coli* O157:H7 protease	*E. coli* O157:H7	[136]
	gold magnetic (Fe_3_O_4_) bifunctional nanobeads	anti-*Salmonella choleraesuis* monoclonal antibodies (_11_D_8_-D_4_ as the detection antibody, _5_F_11_–B_11_ as the capture antibody)	*S. choleraesuis*	[137]
	graphene nanoplatelets	*E. coli* O157:H7-specific antibody	*E. coli* O157:H7	[138]

**Table 5:** Mechanical properties of polymer composites with included cellulose nanoparticles [179].

Mechanical Property	Number of Cellulose Nanoparticles (% (*w*/*w*))	Function
tensile property	5	increase by 42%
water vapor permeability	5	decrease by 28%
oxygen transmission	1	decrease by 21%

## Data Availability

No new data were created or analyzed in this study.
